# Association between Liver Enzymes with Metabolically Unhealthy Obese Phenotype

**DOI:** 10.1186/s12944-018-0847-9

**Published:** 2018-08-23

**Authors:** Junhui Xie, Shujun Zhang, Xuefeng Yu, Yan Yang, Zhelong Liu, Gang Yuan, Shuhong Hu

**Affiliations:** Department of Endocrinology, Tongji Hospital, Tongji Medical College, Huazhong University of Science and Technology, No. 1095 Jiefang Road, Wuhan, 430030 Hubei Province China

**Keywords:** Metabolically unhealthy obesity, Gamma glutamyltransferase, Alanine aminotransferase

## Abstract

**Background:**

Obesity could be classified into two phenotypes: metabolically healthy obesity (MHO) and metabolically unhealthy obesity (MUHO). This study investigated the ability of liver enzymes to identify obesity phenotype.

**Methods:**

We conducted a cross-sectional study in 2197 obese adults (age > 40 years and BMI ≥25 kg/m^2^) in a rural area of central China.

**Results:**

In this population, 75% of the participants have more than one cardiometabolic risk factor. Both GGT and ALT were strongly related to the MUHO phenotype. The association between the fourth quartile of GGT and MUHO risk was strong and independent of confounder risk factors in both genders (adjusted ORs, 1.73 (95%CI 1.03–2.92) for male and 1.82 (95%CI 1.29–2.57) for female). The association between the fourth quartile of ALT and MUHO risk was strong and independent in female, but not in male (adjusted ORs, 1.65 (95%CI 0.86–3.19) for male and 1.88 (95%CI 1.29–2.75) for female). Additionally, AST was not associated with MUHO phenotype.

**Conclusions:**

Both GGT and ALT are effective markers for identifying MUHO in this population. Furthermore, the ability of GGT may be superior to ALT in male.

## Background

The prevalence of obesity is increasing rapidly, which traditionally is characterized by the presence of a cluster of cardiovascular risk factors including insulin resistance, impaired glucose tolerance, atherogenic lipid profiles, and hypertension. However, not all obese people present with metabolic abnormalities. In 1980’s, Andres R et al. suggested that the obese population should be classified into two phenotypes: metabolically healthy obese (MHO) and metabolically unhealthy obese (MUHO) [1]. MHO subjects present beneficial metabolic characteristics, such as lower blood pressure (BP), blood glucose, favorable lipid profiles, insulin sensitivity, ectopic adipose deposition, as well as inflammation [2–6], whereas, the MUHO individuals are on the contrary. In addition, a variety of surveys investigated the incidence of long-term all-cause mortality and cardiovascular events in MHO and MUHO subjects [7]. Surprisingly, all-cause mortality, cardiovascular events and risk of cancer in MUHO were significantly higher than that in MHO group for 5~ 10 years or even 30 years follow-up [8–15]. Therefore, it is important to discriminate the two phenotypes of obesity for diagnostic and therapeutic purpose [16, 17].

Metabolic unhealthy is defined as the presence of the risk factors of metabolic complications, such as hyperglycemia, dyslipidemia, hypertension, central obesity, insulin resistance (IR), inflammation factors or leukocyte [16] basing on ATPIII [18] or IR criterion [19]. It is necessary to explore a simple and effective clinical indicator to identify the MUHO phenotype. The underlying etiology of the MUHO phenotype is not well understood. We hypothesized that hepatic enzyme which is closely associated with liver fat content and hepatic IR may be useful in identifying the MUHO phenotype. A large number of studies showed that liver enzymes including gamma glutamyltransferase (GGT), alanine aminotransferase (ALT) and aspartate aminotransferase (AST) were associated with diabetes mellitus type 2 (T2DM), metabolic syndrome as well as cardiovascular diseases [20–23]. In the present study, we aimed to analyze the association of liver enzymes with MUHO phenotype.

## Methods

### Study sample

The date came from a population-based, cross-sectional study launched in Tianmen District, Hubei Province, which located in the central part of China. In brief, the study was conducted from 2011 to 2012, and the population comprised of residents aged 40 years and older who lived in the local areas for at least 5 years, the subjects distributed in 13 villages in Tianmen District. This study was one part of the Risk Evaluation of cAncers in Chinese diabeTic Individuals: a lONgitudinal (REACTION) study in China [24]. The REACTION study has been set up as a multicenter prospective observational study aiming to evaluate the chronic diseases in Chinese population. We used the data of obese participants with body mass index (BMI) > 25.0 kg/m^2^, according to the Wealth Health Organization criteria for Asians. The exclusion criteria included T2DM, abnormal liver enzymes, heavy alcohol intake (≥40 g alcohol per day), and missing data on age, sex, BP, BMI, and fasting plasma glucose (FPG), insulin, triglyceride (TG), high-density lipoprotein-cholesterol (HDL-C), low-density lipoprotein-cholesterol (LDL-C), GGT, ALT and AST. Altogether, 2197 participants were included in the analysis, including 640 (29.1%) men and 1557 (70.9%) women. All the participation was voluntary without a stipend. The principles outlined in the Declaration of Helsinki (2008) were fully followed. This study is sponsored by the Chinese Society of Endocrinology. All procedures used in this study were in accordance with institutional guidelines [24]. The Committee on Human Research at Tongji Hospital, Tongji Medical College, Huazhong University of Science and Technology, approved the study protocol, and all study participants provided written informed consents.

### Clinical measurements

A standard questionnaire was utilized for demographic characteristics such as age, sex, education level, medical history, smoking history, and current drinking status. Physical examination was performed and anthropometry was obtained, including height, weight, waist circumference (WC), hip circumference (HC), and BP. BMI was calculated as weight (kg)/square of height (m2). BP was measured following standardized protocols by trained examiners using a mercury sphygmomanometer with appropriate cuff at 3 different consecutive times at 3 to 5-min intervals. The 3 readings were averaged as the BP values in our data analysis.

Diabetic history was defined as self-reported current treatment with anti-diabetic drugs. The participants without T2DM underwent a two-hour oral glucose tolerance test (OGTT) with 75 g of glucose and participants with T2DM were given a steamed bun that contained approximately 100 g of complex carbohydrates for safety reasons. Fasting and postprandial 2 h after OGTT blood samples were collected. Serum samples were immediately centrifuged and stored in − 80 °C until analysis. Fasting serum liver enzymes (GGT, ALT, AST), lipid including total cholesterol (TC), TG, HDL-C, LDL-C were measured by the automatic biochemical blood biochemistry detector. Plasma glucose was measured using the hexokinase enzymatic method. Glycosylated hemoglobin (HbA1c) was measured with ion-exchange high-pressure liquid chromatography (HPLC) (glycosylated hemoglobin analyzer, Bio-Rad Variant II, United States). Fasting serum insulin was detected by chemiluminescent microparticle immunoassay. The reference range of GGT is 0–64 U/L in male and 0–36 in female. The reference range of ALT and AST is 0–55 U/L and 5–34 U/L, respectively. The index of homeostasis model assessment of insulin resistance (HOMA-IR) was calculated according to the formula: HOMA-IR = fasting insulin concentration (mIU/L) × fasting plasma glucose concentration (mmol/L)/22.5. All blood samples were evaluated in the central laboratory.

### Definitions

Based on previous studies, we used two definitions to identify metabolic health status: the ATP-III definition of metabolic syndrome [18] and the HOMA-IR index [19].

According to ATP III, the obese subjects who have no less than three following metabolic abnormalities are defined as MUHO and who have less than three following components are defined as MHO: (1) triglycerides ≥1.7 mmol/L or use of lipid-lowering drugs, (2) systolic/diastolic BP ≥130/85 mmHg or use of antihypertensive drugs, (3) fasting glucose ≥5.6 mmol/L or use of medications for diabetes, (4) HDL-C ≤ 1.0/1.3 mmol/L for male/female, and (5) WC ≥90/80 cm for male/female.

According to the definition of HOMA index, the obese subjects who have the highest quartile of HOMA-IR are defined as insulin-resistant obese (OBIR) and who have the lower three quartiles of HOMA-IR are defined as insulin-sensitivity obese (OBIS). The cut-off point of three-quarters of HOMA-IR index is 2.11mIU/L^2^ in men and 2.39 mIU/L^2^ in women.

### Statistical analysis

All statistical analyses were conducted using SPSS version 20.0 (SPSS Inc., Chicago, IL). Continuous variable was expressed as mean ± standard deviation (SD) and categorical variable was presented as percentage. Difference in general feature between groups was assessed using t test.

Binary logistic regression analysis was performed to calculate odds ratio (OR) and 95% confidence intervals (CI) for MUHO in different GGT and ALT quartiles in male and female participants. Three models were applied: model 1 was unadjusted; Model 2 was adjusted for age, drinking history (for male), systolic BP, diastolic BP, HbA1c; Model 3 was adjusted for all variables in model 2 plus HOMA-IR.

A two-tailed *P* value < 0.05 was considered statistically significant.

## Results

### General characteristics of the participants

In this population, the average age was 59.4 ± 9.5 years, and mean BMI and WC was 27.2 ± 1.9 kg/m^2^ and 86.9 ± 7.7 cm, respectively. 93.2% of the population was rural resident. Clinical characteristics of the population stratified by gender were displayed in Table 1. Compared to the female group, male participants were more likely to be older and to have higher levels of WC, diastolic BP, FPG, however, lower fasting insulin, HbA_1_c, TC, and HDL-C levels (*P* < 0.001). There was no significant difference in systolic BP, BMI, TG, LDL-C, and HOMA-IR between both genders. Notably, serum levels of liver enzymes (GGT, ALT, AST) were higher in male than in female.

**Table 1:** Anthropometric and biochemical measures in male and female

N	Total	Male	Female	P
	2197	640	1557	
Age (y)	59.4 ± 9.5	60.7 ± 9.7	58.8 ± 9.4	< 0.000
BMI (kg/m^2^)	27.2 ± 1.9	27.1 ± 1.8	27.3 ± 1.9	0.102
WC (cm)	87.7 ± 7.7	89.5 ± 7.5	86.9 ± 7.7	< 0.000
W/H	1.1 ± 0.9	0.9 ± 0.1	1.3 ± 0.1	< 0.000
SBP (mmHg)	117.3 ± 18.1	118.2 ± 17.5	116.9 ± 18.3	0.132
DBP (mmHg)	75.7 ± 10.7	77 ± 11	75.1 ± 10.6	< 0.000
FPG (mmol/L)	5.3 ± 0.5	5.4 ± 0.5	5.3 ± 0.5	< 0.000
PPG (mmol/L)	6.4 ± 1.5	6.2 ± 1.6	6.5 ± 1.5	0.002
FINS (μU/ml)	8.3 ± 5.9	7.7 ± 7.3	8.5 ± 5.3	0.007
HbA1c(%)	5.8 ± 0.4	5.7 ± 0.4	5.8 ± 0.4	0.001
HOMA-IR	2.0 ± 1.5	1.9 ± 1.9	2.0 ± 1.3	0.09
TG (mmol/L)	1.8 ± 1.1	1.7 ± 1.1	1.8 ± 1.1	0.072
TC (mmol/L)	5.2 ± 1.0	5.0 ± 1.0	5.2 ± 1.0	< 0.000
HDL-C (mmol/L)	1.4 ± 0.3	1.3 ± 0.3	1.4 ± 0.3	< 0.000
LDL-C (mmol/L)	3.0 ± 0.8	2.9 ± 0.8	3.1 ± 0.8	0.057
AST (U/L)	22.9 ± 10.1	24.5 ± 8.0	22.3 ± 10.8	< 0.000
ALT (U/L)	15.9 ± 11.7	18.4 ± 11.8	14.9 ± 11.5	< 0.000
GGT (U/L)	24.2 ± 11.9	30.2 ± 12.8	21.8 ± 10.6	< 0.000

Data are means ± SD

*BMI* body mass index, *WC* waist circumference, *W/H* waist hip ratio, *SBP* systolic blood pressure, *DBP* diastolic blood pressure, *FPG* fasting plasma glucose, *PPG* postprandial 2 h plasma glucose, *FINS* fasting insulin, *HbA1c* glycosylated hemoglobin, *HOMA-IR* FPG × FINS/22.5, *TG* triglycerides, *TC* total cholesterol, *HDL-C* high-density lipoprotein- cholesterol, *LDL-C* low-density lipoprotein- cholesterol, *GGT* γ-glutamyltransferase, *ALT* alanine aminotransferase, *AST* aspartate aminotransferase

*P*-value: comparison between male and female

In our study, 75% of the participants have more than one cardiometabolic risk factor according to the ATPIII definition. The prevalence of the population presenting with two, three or four cardiometabolic risk factors was 30.1%, 26.7%, 15.8% in male, and 31%, 27.8%,18.5% in female, respectively. Only 8.1% males and 3% females presented without cardiometabolic risk factor. Furthermore, the prevalence of central obesity and hypertension ranked the first (31.3%) and second (28.6%) in these cardiometabolic risk factors.

### Comparison of the subgroups defined by two criteria of metabolic health status

The population was divided into MHO and MUHO subgroups according to the ATPIII definition, and into OBIS and OBIR subgroups based on the HOMA-IR definition, respectively. According to ATPIII definition, the prevalence of MUHO was 47.6% in this population. 233 males (36.4%) and 813 females (52.2%) presented MUHO phenotype. Based on the HOMA-IR criterion, the prevalence of OBIR was 25.2% in the population. 163 males (25.5%) and 390 females (25.1%) were OBIR. It seems that the MUHO phenotype was more popular in female than in male, nevertheless, the prevalence of OBIR phenotype was comparative in both genders. Besides, the prevalence of MUHO was higher than that of OBIR either in male or in female.

Furthermore, clinical characteristics of the population stratified by gender and metabolic status were presented in Table 2. Based on ATPIII criteria, compared with MHO subgroup, MUHO subgroup had higher levels of BMI, WC, W/H, BP, TG, TC, FPG, HbA_1_c, HOMA-IR, and fasting insulin, and lower levels of HDL-C, no matter in male or in female**.** According to HOMA-IR index criteria, OBIR subjects had higher levels of BMI, WC, TG, FPG, HOMA-IR, and fasting insulin, and lower levels of HDL-C than OBIS individuals in both genders**.** Concerning liver enzymes, serum GGT and ALT levels were significantly higher in MUHO subgroup than in MHO subgroup in both genders (MHO vs. MUHO, GGT in male: 28.7 ± 12.1 U/L vs. 32.8 ± 13.7 U/L; in female: 20.2 ± 10.0 U/L vs. 23.3 ± 11 U/L; ALT in male: 17.6 ± 12.3 U/L vs 19.8 ± 10.7 U/L; in female: 14.6 ± 13.7 U/L vs 15.2 ± 8.8 U/L). Compared to OBIS group, serum GGT levels were higher in OBIR group of both genders, and serum ALT levels were higher only in OBIR male group (OBIS vs. OBIR, GGT in male: 28.6 ± 12.3 U/L vs. 34.8 ± 13.7 U/L; in female: 20.9 ± 10.2 U/L vs. 24.3 ± 11.2 U/L; ALT in male: 17.3 ± 10.1 U/L vs 21.5 ± 15.3 U/L; in female: 14.4 ± 11.8 U/L vs 16.3 ± 10.5 U/L). There was no statistically significant difference of AST levels between subgroups.

**Table 2:** Comparison of the subgroups defined by the ATPIII and HOMA-IR

	ATPIII				HOMA-IR			
	male		female		male		female	
	MHO(407)	MUHO (233)	MHO(744)	MUHO (813)	OBIS (477)	OBIR(163)	OBIS (1167)	OBIR(390)
Age (y)	61.3 ± 9.9	59.6 ± 9.4^*^	58.0 ± 10.0	59.7 ± 8.8^▲^	61.6 ± 9.2	58.3 ± 10.1^▲^	59.0 ± 9.5	58.5 ± 9.3
BMI (kg/m^2^)	26.8 ± 1.7	27.7 ± 1.9^▲^	27.0 ± 1.8	27.6 ± 1.9^▲^	26.9 ± 1.6	27.9 ± 2.2^▲^	27.0 ± 1.7	28.0 ± 2.1^▲^
WC (cm)	97.0 ± 6.0	101.1 ± 5.5^▲^	84.9 ± 7.9	89.0 ± 7.0^▲^	88.5 ± 7.4	92.4 ± 7.2^▲^	86.4 ± 7.6	88.6 ± 7.8^▲^
W/H	0.9 ± 0.1	0.9 ± 0.1^▲^	0.9 ± 0.5	0.9 ± 0.6^▲^	0.9 ± 0.05	0.9 ± 0.0^▲^	0.9 ± 0.1	0.9 ± 0.1^*^
SBP (mmHg)	115.8 ± 18.6	122.2 ± 14.7^▲^	112.3 ± 18.3	121.5 ± 17.1^▲^	118.4 ± 18.0	117.4 ± 16.0	116.9 ± 18.5	117.0 ± 17.5
DBP (mmHg)	75.1 ± 10.9	80.2 ± 10.5^▲^	72.9 ± 10.1	77.2 ± 10.6^▲^	76.8 ± 11.3	77.6 ± 10.1	74.8 ± 10.7	76.0 ± 10.0
FPG (mmol/L)	5.3 ± 0.5	5.7 ± 0.5^▲^	5.1 ± 4.3	5.5 ± 0.6^▲^	5.4 ± 0.5	5.7 ± 0.6^▲^	5.2 ± 0.5	5.6 ± 0.5^▲^
PPG (mmol/L)	6.0 ± 1.5	6.7 ± 1.7^▲^	6.1 ± 1.4	6.8 ± 1.56^▲^	6.1 ± 1.6	6.6 ± 1.6^▲^	6.4 ± 1.5	6.7 ± 1.6^▲^
FINS (μU/ml)	6.6 ± 4.6	9.5 ± 10.2^▲^	7.4 ± 4.2	9.7 ± 6.0^▲^	5.3 ± 1.7	14.6 ± 11.6^▲^	6.4 ± 2.0	14.9 ± 6.8^▲^
HbA_1_c(%)	5.6 ± 0.4	5.8 ± 0.5^▲^	5.7 ± 0.4	5.9 ± 0.4^▲^	5.7 ± 0.4	5.8 ± 0.5^*^	5.7 ± 0.4	5.9 ± 0.5^▲^
HOMA-IR	1.6 ± 1.2	2.4 ± 2.8^▲^	1.7 ± 1.0	2.4 ± 1.5^▲^	1.3 ± 0.4	3.7 ± 3.1^▲^	1.5 ± 0.5	3.7 ± 1.7
TG (mmol/L)	1.3 ± 0.6	2.4 ± 1.3^▲^	1.3 ± 0.6	2.3 ± 1.3^▲^	1.6 ± 1.0	2.1 ± 1.2^▲^	1.7 ± 1.0	2.1 ± 1.4^▲^
TC (mmol/L)	4.9 ± 0.9	5.2 ± 1.1^▲^	5.1 ± 0.9	5.4 ± 1.1^▲^	5.0 ± 0.9	5.2 ± 1.0	5.2 ± 1.0	5.4 ± 1.0^*^
HDL (mmol/L)	1.4 ± 0.3	1.2 ± 0.3^▲^	1.5 ± 0.3	1.3 ± 0.3^▲^	1.4 ± 0.3	1.3 ± 0.3^▲^	1.4 ± 0.3	1.4 ± 0.3^▲^
LDL (mmol/L)	3.0 ± 0.8	3.0 ± 0.8	3.0 ± 0.7	3.1 ± 0.9^▲^	3.0 ± 0.8	3.1 ± 0.8	3.0 ± 0.8	3.2 ± 0.9^*^
AST (U/L)	24.5 ± 8.5	24.6 ± 6.9	22.9 ± 13.6	21.7 ± 7.0	24.5 ± 7.5	24.5 ± 9.1	22.5 ± 11.6	21.7 ± 7.9
ALT (U/L)	17.6 ± 12.3	19.8 ± 10.7^▲^	14.6 ± 13.7	15.2 ± 8.8^▲^	17.3 ± 10.1	21.5 ± 15.3^▲^	14.4 ± 11.8	16.3 ± 10.5
GGT (U/L)	28.7 ± 12.1	32.8 ± 13.7^▲^	20.2 ± 10.0	23.3 ± 11^▲^	28.6 ± 12.3	34.8 ± 13.2^▲^	20.9 ± 10.2	24.3 ± 11.2^▲^

Data are means ± SD

*BMI* body mass index, *WC* waist circumference, *W/H* waist hip ratio, *SBP* systolic blood pressure, *DBP* diastolic blood pressure, *FPG* fasting plasma glucose, *PPG* postprandial 2 h blood glucose, *FINS* fasting insulin, *HbA1c* glycosylated hemoglobin, *HOMA-IR* FBG × FINS/22.5, *TG* triglycerides, *TC* total cholesterol, *HDL* high-density lipoprotein, *LDL* low-density lipoprotein- cholesterol, *GGT* γ-glutamyltransferase, *ALT* alanine aminotransferase, *AST* aspartate aminotransferase, *MHO* metabolically healthy obese, *MUHO* metabolically unhealthy obese, *OBIS* insulin sensitive obese, *OBIR* insulin resistant obese

^*^P < 0.05,^▲^*P* < 0.001: MHO subgroup vs. MUHO subgroup; OBIS subgroup vs. OBIR subgroup

### The correlations between liver enzymes and cardiometabolic risk factors

We next studied the correlations between liver enzymes and cardiometabolic risk factors in both genders by using Pearson correlation analysis. After adjusting for age, sex, and BMI, GGT was positively associated with TG (*r* = 0.317, *p* < 0.01), HOMA-IR (*r* = 0.174, *p* < 0.01), FPG (*r* = 0.143,*p* < 0.01) and WC (*r* = 0.129, *p* < 0.01), and not associated with BP and HDL-C. Furthermore, ALT had significantly positive correlation with TG (*r* = 0.2, *p* < 0.01) and WC (*r* = 0.089, *p* < 0.05), on contrast, it was inversely associated with HDL-C (*r* = − 0.134, *p* < 0.01) and had no significant relationship with BP and FPG. Besides, there was no significant correlation between AST and all the evaluated risk factors.

### The association between GGT and ALT with MUHO risk

Then, we examined the separate association of ALT and GGT with MUHO in male and female groups, respectively. The ORs for MUHO risk were shown in Tables 3 and 4. The participants were separated into four subgroups (Q1~Q4) according to the quartiles of GGT and ALT respectively. The lowest quartile group (Q1) was taken as the reference.

**Table 3:** Logistic regression analysis of association of GGT and ALT with the risk of metabolic unhealthy according to ATPIII criterion in obese male

	N	Model 1 OR(95%CI)	Model 2 OR(95%CI)	Model 3 OR(95%CI)
		Sig.(P)	Sig. (P)	Sig. (P)
GGT (U/L)				
Q1(≤20)	171	1	1	1
Q2(21–27)	156	1.87(1.17–2.98)	1.59(0.87–2.6)	1.58(0.95–2.63)
		0.009	0.07	0.08
Q3(28–39)	161	1.38(0.86–2.2)	1.05(0.63–1.76)	1.08(0.64–1.82)
		0.19	0.85	0.79
Q4(≥40)	152	2.55(1.6–4.07)	1.82(1.1–3.04)	1.73(1.03–2.92)
		0	0.02	0.04
ALT (U/L)				
Q1(≤12)	200	1	1	1
Q2(13–15)	128	0.87(0.54–1.42)	0.89(0.53–1.49)	0.98(0.56–1.71)
		0.59	0.65	0.94
Q3(16–22)	167	1.32(0.86–2.03)	1.12(0.69–1.8)	1.05(0.62–1.75)
		0.21	0.65	0.87
Q4(≥23)	145	1.88(1.21–2.92)	1.8(1–3.27)	1.65(0.86–3.19)
		0.005	0.05	0.14

Data are described as odds ratios (95%CI)

Model 1 was unadjusted

Model2 was adjusted for age, drinking history, systolic BP, diastolic BP, HbA_1_c

Model 3 was adjusted for all variables in Model 2 plus HOMA-IR

**Table 4:** Logistic regression analysis of association of GGT and ALT with the risk of metabolic unhealthy according to ATPIII criterion in obese female

		Model 1 OR(95%CI)	Model 2 OR(95%CI)	Model 3 OR(95%CI)
		Sig.(P)	Sig.(P)	Sig.(P)
GGT (U/L)				
Q1(≤14)	171	1	1	1
Q2(15–19)	156	1.16(0.88–1.52)	0.97(0.72–1.31)	0.94(0.71–1.31)
		0.29	0.85	0.70
Q3(20–26)	161	1.75(1.31—2.35)	1.27(0.92–1.76)	1.22(0.9–1.74)
		0	0.15	0.25
Q4(≥27)	152	2.35(1.74–3.11)	1.73(1.24–2.41)	1.82(1.29–2.57)
		0	0	0.001
ALT (U/L)				
Q1(≤10)	486	1	1	1
Q2(11–13)	379	1.45(1.1–1.9)	1.39(1.04–1.87)	1.38(1.02–1.88)
		0.007	0.03	0.04
Q3(14–17)	330	1.86(1.4–2.46)	1.8(1.31–2.47)	1.82(1.32–2.52)
		0	0	0
Q4(≥18)	364	1.7(1.29–2.24)	2.02(1.4–2.93)	1.88(1.29–2.75)
		0	0	0.001

Data are described as odds ratios (95%CI)

Model 1 was unadjusted

Model2 was adjusted for age, drinking history, systolic BP, diastolic BP, HbA_1_c

Model 3 was adjusted for all variables in Model 2 plus HOMA-IR

For male, the unadjusted ORs for MUHO (model 1) were 1.87 (1.17–2.98), 1.38 (0.86–2.2), 2.55(1.6–4.07) among subjects in the second, the third, and the fourth quartile of GGT, as compared with persons in the first GGT quartile. After adjustment for the potential confounding, the ORs for MUHO were decreased but remained significant only in the fourth quartile of GGT (1.73 (1.03–2.92)). For female, we observed a trend of increasing ORs for MUHO with increasing quartiles of GGT in all models. The unadjusted ORs of the second, the third, and the fourth quartile of GGT were 1.16 (0.88–1.52), 1.75(1.31–2.35) and 2.35(1.74–3.11), respectively. After adjusting for potential risk factors, the ORs remained significant only in the fourth quartile of GGT (1.82 (1.29–2.57)).

As for ALT, the crude ORs for MUHO were 0.87 (0.54–1.42), 1.32(0.86–2.03) and 1.88(1.21–2.92) for the increasing quartiles of ALT in male. We observed that only the fourth quartile of ALT was significantly positively associated with the MUHO risk, and the ORs gradually decreased after adjusted for confounder risk factors (model 2), however, the association was still statistically significant (OR, 1.8 (95%CI 1–3.27)). Furthermore, when HOMA-IR were further adjusted in model 3, the OR of the fourth ALT quartile was 1.65 (0.86–3.19) which showed no statistical significance. In female, ALT showed a strong association with MUHO phenotype. In model 1, the crude ORs for MUHO risk of the second to fourth ALT quartiles were 1.45 (1.1–1.9), 1.86 (1.4–2.46), and 1.70 (1.29–2.24), respectively. After adjustment for the potential confounding (model 3), the ORs of the second to fourth quartiles were 1.38 (1.02–1.88), 1.82 (1.32–2.52) and 1.88 (1.29–2.75), respectively, which remained statistically significant.

## Discussion

In this population, we observed that 75% of the participants have more than one cardiometabolic risk factor. Both GGT and ALT were associated with the MUHO phenotype. Of note, the association between the fourth quartile of GGT and MUHO risk was close and independent of confounder risk factors in both genders (adjusted ORs, 1.73 (95%CI 1.03–2.92) for male and 1.82 (95%CI 1.29–2.57) for female), nevertheless, the association between the fourth quartile of ALT and MUHO risk only existed in female, but not in male (adjusted ORs, 1.65 (95%CI 0.86–3.19) for male and 1.88 (95%CI 1.29–2.75) for female).

Our results suggested that both GGT and ALT were associated with MUHO phenotype. Our findings are consistent with the role of liver fat in MUHO pathogenesis. Recently, both ALT and GGT, even within the normal range, have been reported to predict incident diabetes, nonalcoholic fatty liver disease, or other metabolic disorders [25, 26]. It was demonstrated that the correlations between GGT and ALT with cardiovascular risks were attributed to oxidative stress, inflammation and IR [27]. Moreover, the visceral fat were significantly larger in MUHO than in MHO [24]. There has been reported that the ectopic fat deposition (especially in liver) in MHO decreased 53% compared with MUHO [6]. Because fat deposition in liver induces IR, inflammation and further metabolic disorders, finally the fat easily infiltrated into the other organs (such as skeletal muscle, visceral fat tissue, myocardial) [28]. Higher levels of liver enzymes may indicate higher liver fat deposition, and IR in liver is more severe in MUHO subjects, which partly explained the mechanism of the MUHO phenotype. Although CT or MRI is an ideal measurement of visceral fat deposition in obese population, liver enzymes are more convenience and cheaper indicators.

Our data revealed that GGT may be a better predictor for MUHO risk than ALT, especially in male. Our observation was consistent with a meta-analysis which has demonstrated a stronger association between GGT and diabetes than between ALT and diabetes [29]. There are two possible explanations for the potentially stronger association of GGT with MUHO risk. First, although both ALT and GGT are biomarkers of liver fat, GGT may be the better marker [29]. This explanation cannot be ruled out in light of the paucity of relevant evidence as to which liver enzyme better reflects liver fat content. On the other hand, GGT is present and highly active in organs other than the liver, such as the kidney and pancreas [30], thus, ALT is considered a more specific marker of liver fat content than GGT. However, GGT is the enzyme responsible for the extracellular catabolism of antioxidant glutathione [31], and it may be linked to greater oxidative stress which has been implicated in insulin resistance, diabetes, and cardiovascular disease [32, 33]. Presumably, GGT’s potentially stronger association with metabolic disorders may be attributed to its involvement in several different processes relevant to the pathogenesis of MUHO. Besides, this finding was mainly present in male group, while in female group GGT and ALT were comparative in association with MUHO. The underlying mechanism remains unclear. Future studies are required to testify the suggestion.

As we all know, AST is not as sensitive or specific to liver damage as ALT and GGT [34]. Consistently, AST has also been assessed in our data, but it seems to be not relevant to cardiometabolic risk factors. Therefore, the role of AST was not addressed in our study. In addition, the present study showed the risk of MUHO significantly increased with GGT levels independent of IR even for subjects in normal range of GGT for both genders, while the MUHO risk increased with increasing ALT only in female group. Thus, GGT seems to be a potentially better diagnostic indicator of MUHO among the three liver enzymes. Notably, in a Brazilian study [35], the mean value of GGT in metabolically unhealthy overweight/obese individuals was reported to be 52 U/L, which was higher than the level of GGT in our study. This may be attributed to the exclusion of the individuals with abnormal GGT in our study, whereas the Brazilian study included the whole population.

There were some limitations in our survey. Firstly, the majority of the sample was partially representative rural resident in China, thus, extrapolating results to the whole Chinese people and other population should be interpreted cautiously. Secondly, the subjects included the non-diabetic participants with the normal range of liver enzymes so that the incidence of substantial liver steatosis and other hepatic diseases was low. Further cohort study would be necessary to investigate the association between liver enzymes and cardiometabolic risk in the obese population.

## Conclusions

In conclusion, GGT and ALT were associated with MUHO risk above and beyond commonly measured metabolic risk factors. Our results suggested that the association of GGT with MUHO risk may be of greater magnitude than that of ALT with MUHO, especially in male. Serum GGT is convenient to detect and inexpensive item especially as the evaluation of the cardiometabolic risk in epidemic survey. We recommend serum GGT as regular item screening for the metabolically unhealthy risk in even the obese individual with normal range of GGT.

## Abbreviations

ALT: Alanine aminotransferase
AST: Aspartate aminotransferase
BMI: Body mass index
BP: Blood pressure
CI: Confidence intervals
FPG: Fasting plasma glucose
GGT: Gamma glutamyltransferase
HbA1c: Glycosylated hemoglobin
HC: Hip circumference
HDL-C: High-density lipoprotein-cholesterol
HOMA-IR: Homeostasis model assessment of insulin resistance
IR: Insulin resistance
LDL-C: Low-density lipoprotein-cholesterol
MHO: Metabolically healthy obese
MUHO: Metabolically unhealthy obese
OBIR: Insulin-resistant obese
OBIS: Insulin-sensitivity obese
OGTT: Oral glucose tolerance test
OR: Odds ratio
SD: Standard deviation
T2DM: Diabetes mellitus type 2
TC: Total cholesterol
TG: Triglyceride
WC: Waist circumference
